# Case of metachronous clear cell bladder metastasis on low risk localized renal cell carcinoma patient

**DOI:** 10.1016/j.ijscr.2022.107158

**Published:** 2022-05-06

**Authors:** Mahdy Alief Adhiguna, Sawkar Vijay Pramod, Bambang Sasongko Noegroho, Ferry Safriadi, Bethy Suryawathy Hernowo

**Affiliations:** a Department of Urology, Hasan Sadikin Academic Medical Center - Faculty of Medicine, Universitas Padjadjaran, Bandung, Indonesia; b Department of Anatomical Pathology, Hasan Sadikin Academic Medical Center - Faculty of Medicine, Universitas Padjadjaran, Bandung, Indonesia

**Keywords:** Case report, Metachronous bladder cancer, Renal cell carcinoma, Bladder metastasis

## Abstract

**Introduction:**

Renal cell carcinoma (RCC) is well known for its ability to metastasize into different organs. However, the management of metachronous RCC is still not yet standardized.

**Case Presentation:**

A 62 years old man was presented with haematuria for the last 2 months. CT scan revealed bladder mass with a size of 2,5 cm and underwent en-bloc resection of bladder mass. The histopathological result showed non-muscle-invasive bladder clear cell renal carcinoma. The patient had a history of left nephrectomy in 2017 and meningioma mass metastasectomy in 2020 with the same histopathological origin.

**Conclusion:**

Bladder metastasis of RCC can be treated by endoscopic surgical intervention.

## Highlights

• Renal cell carcinoma (RCC) is well known for its ability to metastasize into different organs.
• A 62 years old man was presented with a haematuria for the last 2 months.
• The histopathological result showed non-muscle-invasive bladder clear cell renal carcinoma.
• Bladder metastasis of RCC can be treated by endoscopic surgical intervention.

## 1 Introduction

Renal cell carcinoma (RCC) accounts for approximately 5% of adult cases of cancer in men and 3% in women and is the second most common urologic neoplasm. An estimated 18% of patients with RCC have metastasis at diagnosis (synchronous metastasis), and more than 50% will develop metastatic RCC after nephrectomy during follow-up (metachronous metastasis) [1]. The risk of developing a metachronous tumor is greater when there is a family history of RCC, multifocal first renal cell carcinoma, and young patient age, or if the patient has a genetic cancer syndrome such as Von Hippel-Lindau disease [2].

Renal cell carcinoma can metastasize to nearly every organ, although metastatic spread to the urinary bladder is rare, with fewer than 70 described cases around the world [3]. It is also reported that in about 8% of RCC cases, it tends to metastasize to the brain [4]. In this report, we present a patient who developed primary renal carcinoma and metachronous bladder and brain. Based on our knowledge, this combination of multiple carcinomas has never been reported in the literature.

The work has been reported in line with the SCARE criteria [5].

## 2 Case presentation

A 62 years old man was presented with a chief complaint of hematuria for the last 2 months. There was no history of gross hematuria or malignancy in the patient's family. Physical findings were within the normal limit. Kidney functions were within normal limits with a serum creatinine of 0.5 mg/dL and blood urea of 10 mg/dL. Urinalysis was conducted and revealed a great number of red blood cells.

**Fig. 1:**
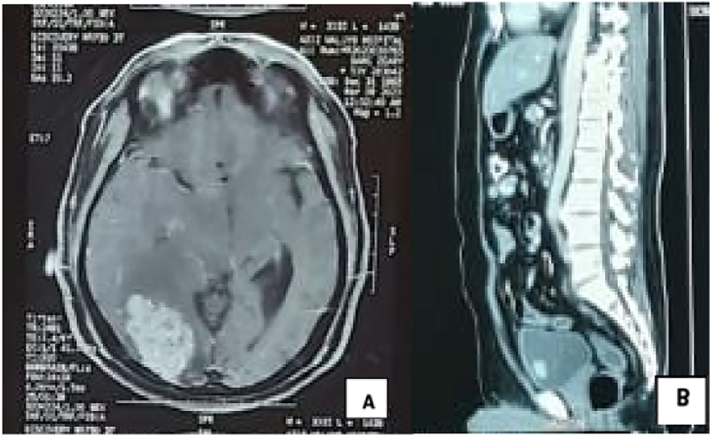
(A) Computed Tomography Scan of the brain showing meningioma, and (B) bladder mass found in CT Scan Imaging.

**Fig. 2:**
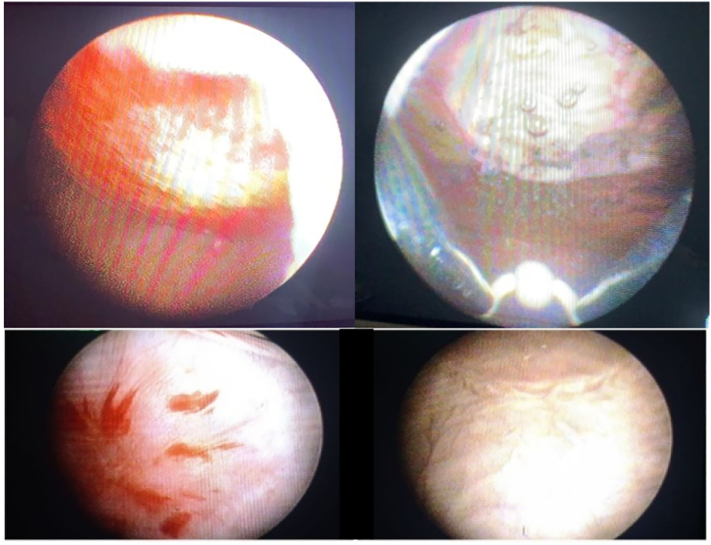
(A) Endoscopic view of bladder tumor; (B) bladder tumor after en-bloc resection; (C) scar tissue found after months post-operation.

**Fig. 3:**
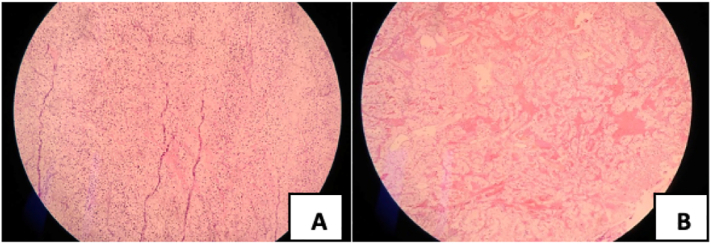
(A) Histopathological renal cell carcinoma in left kidney showing the same pattern of clear cell with (B) clear cell renal carcinoma on bladder without invasion of the detrusor muscle shown by arrows.

**Fig. 4:**
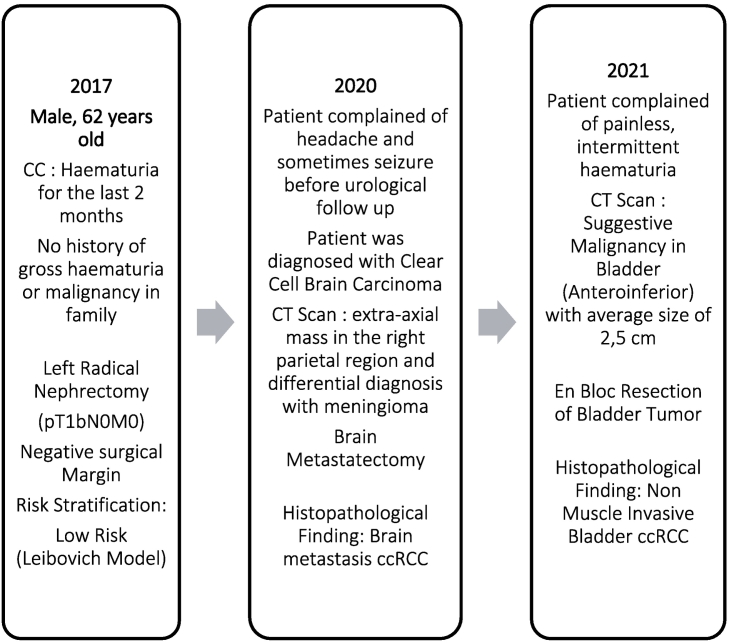
Timeline of patient's medical history.

The patient had been diagnosed with clear cell carcinoma in the left kidney and underwent a left nephrectomy in 2017. Macroscopically, the mass found in the kidney was a soft, brownish, and well-defined lump with a size of 7 cm. Histopathological examination showed dense round cell proliferation with clear cytoplasm with negative surgical margin and a well-defined pseudocapsule. The risk stratification for this patient was low risk according to the Leibovich model. (See Fig. 1, Fig. 2, Fig. 3, Fig. 4.)

In 2020, the patient complained of chronic headaches and infrequent seizures. MRI Brain with contrast revealed an extra-axial mass in the right parietal region with wide perifocal edema in the right cerebral hemisphere and subfalcine herniation, which we differential diagnosed with meningioma. The patient had metastasectomy afterward, and histopathological findings showed metastases clear cell renal carcinoma in meninges.

In 2021, the patient complained of painless, intermittent, reddish urine. The patient underwent cystoscopy that showed a sessile mass on the anterior bladder wall. Abdominopelvic CT scan showed a mass of 2.5 × 2.5 cm on the anterior bladder wall. The patient underwent en-bloc resection of bladder tumor with histopathology examination revealed clear cell renal carcinoma without invasion to the detrusor muscle. In the follow-up cystoscopy, we found scar tissue at the location of the previously resected tumor.

## 3 Discussion

Renal cell carcinoma (RCC) is the second most common urologic neoplasm and accounts for approximately 5% of adult cases of cancer in men and 3% in women. The metastatic disease occurs in a vast majority of RCC patients. An estimated 18% of patients with RCC were found with synchronous metastasis, and more than 50% were found to have developed metachronous metastatic RCC during follow-up [1]. Furthermore, there is a 7% chance of metachronous metastatic disease up to 5 years after nephrectomy and a 16% chance at 10 years. Patients with clear cell RCC, the most common subtype of RCC, have the highest risk of developing metastatic disease. Over 90% of metastatic RCC patients are diagnosed pathologically as clear cell RCC, while the rest is in a heterogeneous group of non-clear cell RCC subtypes, including papillary, chromophobe, collecting duct, and unclassified RCC [6], [7]. Most common sites of metastasis begin with the lungs (40.6%), followed by retroperitoneal space (15.8%), bone (11.9%), lymph node (5.0%), liver (4.0%), brain (1.0%) and other organs (21.8%) [7]. Metastatic spread to the urinary bladder is rare, with fewer than 70 described cases [3].

The underlying mechanism of metastatic spread of RCC is still a dubious topic of discussion, the first pathway is ‘drop metastasis’ where the tumor cells are directly extended and implanted down the urinary tract into the ureter and urinary bladder [8]. Other routes comprise the direct hematogenous route, lymphatic spread, or retrograde venous route where the embolism of tumor cells from the renal vein into the numerous venous connections of the left renal vein [8]. Another theory of tumor metastasis, known as hematogenous metastasis, implicates that invasion of other organs was achieved through tumor cells penetrating blood vessels via the general circulation [8]. Since our patient had multiple sites of metastasis, including the bladder and brain, our case suggests two mechanisms of metastasis, hematogenous metastasis is responsible for RCC metastasis to the brain and drops metastasis to the bladder. Genomic studies identifying the genes for kidney cancer, including the VHL, PBRM1, MET, FLCN, fumarate hydratase, succinate dehydrogenase, TSC1, TSC2, and TFE3 genes are known to play role in renal cell carcinoma development.

Clear cell carcinoma of the bladder is an uncommon malignancy in the urinary tract, that usually affects women with clinical presentation of haematuria and lower urinary tract symptoms (LUTS). Macroscopically, clear cell carcinoma of the bladder can be seen as a papillary and sessile lesion while microscopically the cells are characterized as cuboidal or flat neoplastic cells with hobnailing and abundant eosinophilic cytoplasm or clear cells [3]. Although considered rare, clear cell carcinoma of the bladder could be a result of metastasis from prior clear cell renal cell carcinoma [3].

Studies show patients who underwent radical nephrectomy before, develop gross haematuria later in life that is diagnosed as bladder metachronous RCC, in addition to other sites of metastases such as adrenal, liver, prostate, and thyroid. These patients' age ranged from 55 to 80 years old and were later managed by metastasectomy, targeted therapy, chemotherapy, and even palliative care [3], [9].

The brain itself accounts for approximately 8% of all metastasis RCC cases, with prominent symptoms such as headache, seizure, or decreased mental status. Many show poor prognosis with poor quality of life [4].

Metastasectomy is one of the preferred therapy along with systemic targeted therapy such as sunitinib and local targeted stereotactic radiosurgery that produced good responses in several patients, although there is no absolute consensus at present on patient treatment selection, given the rarity of the case [9]. (See Table 1.)

**Table 1:** Research on the topic of urinary bladder involvement of metastatic clear cell RCC.

Reference	Year	Age	Symptoms	Past history	Management	Metastasis	Nationality
Melegari et al	2010	65	Hematuria	TURBT	Left radical nephrectomy, lymphadenectomy	Renal, bladder, prostate	Italy
De Groote et al	2017	80	Gross hematuria	Left Radical nephrectomy	Targeted therapy	Renal, bladder, liver	Belgium
Menon et al	2015	72	LUTS, hematuria	Left radical nephrectomy	Palliative care	Renal, bladder, ureter	India
Babar et al	2019	79	Urinary retention, gross hematuria	Left radical nephrectomy	Chemotherapy	Renal, bladder, thyroid	USA
Wang et al	2015	55	Hematuria	Left Radical nephrectomy	Adrenalectomy, TURBT, Targeted therapy	Renal, bladder, adrenal gland	China

There were several studies with urinary bladder metastasis of clear cell RCC. De Groote et al. with their research regarding metachronous metastatic ccRCC to the bladder stated that solitary bladder lesion that developed in a metachronous fashion is better than those with synchronous and having multiple metastases, on their research, the patient underwent targeted therapy [6]. Menon et al. found that clinical presentation of haematuria and bladder mass with a history of renal malignancy, and papillary RCC must also be considered. In another research, Babar et a stated that renal cell carcinoma has been shown to unusually metastasize to the urinary bladder, a rarely reported organ of metastasis. Treatment options, such as immunotherapy, are available to patients with such metastasis and long-term survivorship can be achieved. Antonelli et al. considered that the presence of predisposing factors to metachronous RCC should lean forward to conservative management opting for active surveillance (AS), or less invasive procedures like ablation therapy.

## 4 Conclusion

Metachronous renal cell cancer is a rare case in both the urology and oncology field. Multiple primary cases are also rare cases that occurred with brain metastasis and bladder as one rare entity that occurred. Thus, urinary bladder metastasis from renal cell carcinoma is a rare condition that is hardly found.

The mechanism of spread to the urinary bladder is still unclear. Several investigators have proposed direct extension and implantation. Many suggested that venous embolism of tumor cells from the renal vein into its venous connections since there were some findings of left-sided renal tumors that lead to the bladder. Drop metastases are most likely to be the mechanism of our patient pathological condition. Although there are many options for treating urinary bladder metastasis of renal carcinoma, surgical resection could be one of the best options which give satisfactory results.

## Source of funding

None.

## Ethical approval

Ethical approval has been given by Immanuel Hospital's ethical committee.

## Consent

Written informed consent was obtained from the patient for publication of this case report and accompanying images. A copy of the written consent is available for review by the Editor-in-Chief of this journal on request.

## Author's contribution

All authors contribute equally in preparation, concepting, and writing of this manuscript.

## Guarantor

Sawkar Vijay Pramod.

## Provenance and peer review

Not commissioned, externally peer-reviewed.

## Registration of research studies

Not applicable.

## Declaration of competing interest

No potential conflict of interests relevant to this paper was reported.
